# Considerations about the effectiveness and cost effectiveness of therapies in the treatment of hyperphosphataemia

**DOI:** 10.1186/2191-1991-1-1

**Published:** 2011-07-20

**Authors:** Thomas Plagemann, Anne Prenzler, Thomas Mittendorf

**Affiliations:** 1 herescon gmbh - health economic research & consulting, Hannover, Germany; 2 Leibniz University Hanover, Center for Health Economics, Hannover, Germany

## Abstract

Because of an elevated serum phosphate level, patients who suffer from chronic kidney failure frequently tend to have cardiovascular calcification and are therefore exposed to a higher probability of a fatal event. Phosphate binders are able to reduce these negative effects. Currently, there are primarily two groups of phosphate binders (calcium-containing and calcium-free phosphate binders) which are considered to be almost equally effective in terms of binding of free phosphate. There are, however, a few disadvantages of the two groups. While the calcium-containing binders are associated with an increased risk of hypercalcemia, which is dose dependent, calcium-free binders have been criticized as being too expensive. As the expenditure for patients suffering from chronic kidney failure increases from year to year, as a result of increasing prevalence, there is a growing need for an alternative to existing phosphate binders. The study presented here therefore summarizes available information for the novel combination preparation OsvaRen^® ^(calcium acetate/magnesium carbonate) as an alternative therapy to the calcium-free phosphate binder Renagel^® ^(sevelamer-hydrochloride) and to calcium-containing preparations.

The results of this systematic review showed that OsvaRen^® ^is at least equally effective in the regulation of serum phosphate level as Renagel^®^. In particular, OsvaRen^® ^shows no clinically relevant difference in terms of the control of the serum calcium levels compared to Renagel^® ^and thereby does not increase the risk of a hypercalcaemia, in contrast to pure calcium-based phosphate binders. On the other hand, Renagel^® ^therapy is much more frequently associated with gastrointestinal side-effects, a tendency to result in higher tablet burden for patients and high medication costs. The CALMAG study showed that OsvaRen^® ^was at least as effective and safe in terms of controlling serum phosphate and serum calcium levels as Renagel^® ^while, at the same time, resulting in about 80% lower costs. In addition, OsvaRen^® ^offers a lower risk of hypercalcaemia and associated subsequent costs and is thereby also superior to pure calcium-containing phosphate binders.

Because of the effectiveness and tolerability of calcium acetate/magnesium carbonate, OsvaRen^® ^offers a clinically suitable and, at the same time, cost-effective therapeutic option in the therapy of hyperphosphataemia.

## Introduction

About 1.8 million people worldwide with chronic kidney failure undergo dialysis of which almost 90% undergo haemodialysis [1,2]. In the case of long-lasting insufficiency, the kidneys are not able or are not sufficiently able to purify the blood of the breakdown products of the protein metabolism (amongst others urea, creatinine and uric acid). Also, the regulation of electrolytes, water, as well as the acid-base balance is disturbed. In long-term patients, a reduced ability to excrete phosphate therefore frequently results in a rise in the serum phosphate levels [3].

Even modern dialysis procedures cannot completely eliminate this excess phosphate. At the same time, untreated hyperphosphataemia is responsible, amongst other things, for the increased calcification of the blood vessels and the tissue, so that dialysis patients have a highly increased risk of serious cardiovascular disorders (perfusion disorders, cardiovascular events, strokes etc.). Overall, they have a higher morbidity and mortality risk. Consequently, in the USA, the increased serum phosphate level in dialysis patients contributes to a 20 to 25% greater death rate as a result of cardiovascular disorders. For this reason, patients who are in an advanced stage of renal insufficiency are given a phosphate binder in addition to dialysis therapy [4,5].

In Germany, the prevalence of chronic kidney failure in 2006 was around 92,000, of which about 73% of patients underwent dialysis [6]. The phosphate binders available on the market differ considerably. In addition to the differences in composition and mode of action of the different compounds, the effectiveness, tolerability, and the price of the various products differ to a great extent. For this reason, looking at the available therapy options in the area of phosphate binders from a health economic viewpoint is of particular importance.

## Classification of phosphate binders

In practice, different therapies are used to reduce the serum phosphate levels, all of which have the common goal of reducing the phosphate supplied by food. The commonly acknowledged triad of successful treatment consists of low phosphate diet, adequate dialysis, as well as drug therapy using phosphate binders. These substances bind the phosphate in the intestine, so that it can then be excreted from the body via the gastrointestinal tract, instead of reaching the blood. The first phosphate binder came on the market in Germany already in the 1960s. Since then, new preparations have continuously appeared [4].

Generally, a distinction is made between two different classes of phosphate binders:

• Calcium-containing phosphate binders

◦ Calcium carbonate (e. g. Calciumacetat-Nefro^®^, Calciumacetat Prorenal AM^®^, Phos-Ex^®^)

◦ Calcium acetate (e. g. CC-Nefro^®^, Dreisacarb^®^, Calci-Gry^®^)

• Calcium-free phosphate binders

Aluminum salts (e. g. Phosphonorm^®^)

◦ Sevelamer hydrochloride (Renagel^®^)

◦ Sevelamer carbonate (Renvela^®^)

◦ Lanthanum carbonate (Fosrenol^®^)

With the introduction of OsvaRen^® ^(calcium acetate/magnesium carbonate) as a combination preparation, a new class of phosphate binder was added.

Differences between the different phosphate binders exist with respect to the clinical efficacy, possible side-effect profiles, as well as market prices. In the following, the most popular phosphate binders (classes) are characterized and their respective advantages and disadvantages are highlighted. However, already at this point, it might be stated that the ideal preparation which, at the same time, is effective, tolerated, and also cost-effective does not currently exist. A summary is presented in tables 1 and 2.

**Table 1:** Summary of the advantages and disadvantages of different phosphate binders

Phosphate binder	Advantages	Disadvantages (limiting factors)
**Calcium acetate** (e. g. Calcium acetate Nefro^®^, Calcium acetate Prorenal AM^®^, Phos-Ex^®^, amongst others)	• Effectiveness independent of pH value • Lower calcium uptake compared to calcium carbonate • Moderate tablet burden • Highly cost-effective • Good tolerability	• Gastrointestinal complaints • Risk of hypercalcaemia • In some circumstances, influence on vascular calcification

**Calcium carbonate** (e. g. CC-Nefro^®^, Dreisacarb^®^, Calci-Gry^®^, amongst others)	• Effective • Highly cost-effective • Good tolerability	• Effectiveness influenced by pH value • Gastrointestinal complaints • Risk of hypercalcaemia • In some circumstances, influence on vascular calcification

**Aluminum salts** (e. g. Phosphonorm^®^)	• High effectiveness independent of pH value • Highly cost-effective	• High risk of aluminum toxication including encephalopathy and bone diseases • Difficulties in relation to precise dosing • Greater amount of controls

**Sevelamer** (Renagel^®^, Renvela^®^)	• Effective • No risk of hypercalcaemia • Amongst other things, preventative action in relation to calcification and cholesterol	• Frequent gastrointestinal complaints • High costs • High tablet burden, poor compliance • In some circumstances, negative influence on other medication (e.g. binding of fat-soluble vitamins)

**Lanthanum carbonate** (Fosrenol^®^)	• Effectiveness independent of pH value • No risk of hypercalcaemia • Low dosage - low tablet burden, improved compliance	• High costs • In some circumstances, lanthanum particles can be reabsorbed from the intestine and reach the organs

**Combination preparation** (OsvaRen^®^)	• Effectiveness independent of pH value • Lower calcium uptake and reduced risk of hypercalcaemia compared to calcium acetate and calcium carbonate • Good tolerability • Moderate tablet burden • Moderate costs • In some circumstances, preventative effect in relation to calcification	• Monitoring of the magnesium level • In some circumstances, moderate increase in the serum magnesium level

**Table 2:** Brief summary of the different classes of phosphate binders

Property	OsvaRen^®^	Renagel^®^/Renvela^®^	**e.g. CC-Nefro^®^**, Dreisacarb^® ^
**Active compound class**	Phosphate binder with reduced calcium content and proportion of magnesium (combination preparation)	Calcium-free phosphate binder	Calcium-containing phosphate binder

**Active compound**	Calcium acetate/magnesium carbonate	Sevelamer hydrochloride/ Sevelamer carbonate	Calcium acetate Calcium carbonate

**Licensed**	October 2007	April 2001/June 2009	Since the late 1980s

**Brand name**	**OsvaRen**^® ^film tablets	**Renagel**^® ^800 mg film tablets	**Calcium acetate:** Calciumacetat-Nefro^®^, Calciumacetat Prorenal AM^®^, Phos-Ex^®^, amongst others **Calcium carbonate:** CC-Nefro^®^, Dreisacarb^®^, Calci-Gry^®^, amongst others

**Manufacturer**	Fresenius Medical Care	Genzyme Corporation	Various

**Price/Packaging unit **(Germany)	**€ 49.75 **[23]	**€ 297.29 **[23]	• Calciumacetat-Nefro^® ^€ **22.68**, • Calciumacetat Prorenal AM^® ^**€ 18.10**, • Phos-Ex^® ^**€ 27.69**, • CC-Nefro^® ^**€ 24.53**, • Dreisacarb^® ^**€ 24.53**, • Calci-Gry^® ^**€ 24.57**

**Dosing** (Film tablets each day depending on the serum phosphate level)	**3-10**	**3-15**	• Calciumacetat-Nefro^® ^**9-16**, • Calciumacetat Prorenal AM^® ^**9-16**, • Phos-Ex^® ^**8-16**, • CC-Nefro^® ^**12-18**, • Dreisacarb^® ^**6-12**, • Calci-Gry^® ^**1-2**

**Price/day **(Euro)	**€ 0.83 **to **€ 2.76**	**€ 4.95 **to **€ 24.77**	• Calciumacetat-Nefro^® ^**€ 1.02-1.82**, • Calciumacetat Prorenal AM^® ^**€ 0.82-1.45**, • Phos-Ex^® ^**€ 1.11-2.22**, • CC-Nefro^® ^**€ 1.48-2.21**, • Dreisacarb^® ^**€ 0.74-1.48**, • Calci-Gry^® ^**€ 0.25-0.50**

**Treatment cost/year **(Euro)	**€ 300 **to **€ 1,000** on average **€ 650**	**€ 1,800 **to **€ 9,000** on average **€ 5,400**	• Calciumacetat-Nefro^® ^**€ 400-700**, • Calciumacetat Prorenal AM^® ^**€ 300-500**, • Phos-Ex^® ^**€ 400-800**, • CC-Nefro^® ^**€ 500-800**, • Dreisacarb^® ^**€ 300-500**, • Calci-Gry^® ^**€ 100-200**

## Calcium-containing phosphate binders

This group comprises calcium acetate and calcium carbonate. These compounds are considered to be an effective as well as cost-effective therapy option and are therefore the most widely accepted approach for reducing the level of phosphate in haemodialysis. Compared to calcium carbonate, calcium acetate is considered to be more effective in the binding of phosphate in the intestine. Hypercalcaemia and gastrointestinal complaints are the most frequent side-effects caused by treatment with calcium-containing phosphate binders. Gastrointestinal side-effects occur much more rarely, however, in treatment with calcium-containing phosphate binders than in treatment with sevelamer. However, concerns have been raised in relation to usual calcium-containing phosphate binders as they might cause hypercalcaemia. The link with hypercalcaemia is based on the finding that, in particular in combination with vitamin D analogues, the calcification process can be additionally amplified in the vascular system. However, the evidence for this link is not conclusive as two comprehensive meta-analyses carried out by Navatheen et al (2009) [7] and Tonelli et al (2010) [8] were not able to show a significant correlation between sevelamer and a lower cardiovascular calcification as well as lower mortality. For example, the CARE-2 study [9] showed that with a constant LDL level there was no significant difference in the progression of cardiovascular calcification between patients who received a calcium-containing phosphate binder and patients who received sevelamer. In contrast, the authors found that calcium-based phosphate binders, in clinical "head-to-head" study settings, seem to be more effective than sevelamer in reducing the phosphate levels. Raised levels of serum phosphate demonstrably represent the most important independent risk of calcification and mortality in dialysis patients. Apart from calcium acetate and calcium carbonate, there are only a few calcium-containing phosphate binders all of which are rarely used [4,7-10].

## Calcium-free phosphate binders

### Aluminum

Examples of this group are algedrate and the aluminum chloride hydroxide complex. Aluminum-containing phosphate binders are, as a basic rule, very effective but are only rarely used as they are rather toxic. When used on a long-term basis, they cause lingering aluminum intoxication which can be responsible for the inhibition of various enzyme activities. Distortion of perception, disorders of bone metablism (osteomalacias), as well as anaemia is a frequent consequence of long-term application. Clinical symptoms are diagnosed, even with low dosing regimens, so that safe dosing is difficult and use is associated with a high expenditure in terms of the need for control visits [4].

### Sevelamer

Sevelamer hydrochloride is the only synthetically produced aluminum-free and metal-free phosphate binder that mainly binds phosphate by means of ion exchange and hydrogen binding in the duodenum. Sevelamer hydrochloride has been marketed since 1998 in the US and Europe under the brand name Renagel^® ^(Genzyme Corporation). Numerous studies have shown that sevelamer is equally effective in phosphate binding as calcium preparations, but in so doing triggers fewer hypercalcaemic episodes. Moreover, it is suspected that sevelamer is capable of reducing calcification of the heart, as well as the aorta and, in addition, lowering total cholesterol and LDL-cholesterol. Clinical studies of sevelamer have indeed been able to establish a lower rate of hospitalization (as a secondary outcome parameter). However, it has not been possible to demonstrate reduced rates of mortality or morbidity (as a primary outcome). As sevelamer hydrochloride is more costly than calcium-containing compounds and patients have to take relatively more tablets widespread use of this therapeutic option is limited. It is additionally suspected that sevelamer adversely affects the action of other drugs in that these are also bound by the polymer and this thereby reduces the effect of concomitant medications. A new formulation of sevelamer, sevelamer carbonate, was licensed in 2009 under the brand name Renvela^®^. Treatment with sevelamer carbonate appears to cause less acidosis than treatment with sevelamer hydrochloride [4,6-8,11].

### Lanthanum carbonate

Lanthanum carbonate (Fosrenol^® ^from Shire Pharmaceuticals) is an aluminum-free and calcium-free phosphate binder which has been available since 2005 in the US and since 2006 in Europe. Based on evidence gained up until now, it has the same efficacy with respect to the phosphate binding properties as do aluminum-containing preparations, but without the explicit risk of associated intoxication. Although numerous long-term studies have produced no evidence of side-effects, it is nevertheless suspected that lanthanum particles are reabsorbed via the intestine and migrate from there out into the tissues of the organism. Lanthanum carbonate is already very effective in small dosages (normally one tablet per meal), whereby patients experience a comparatively low tablet burden, which is conducive for therapy adherence. However, lanthanum carbonate comes with comparatively high costs, as lanthanum is a rare and therefore expensive metal [4,8].

## Phosphate binders with magnesium and reduced calcium content

OsvaRen^® ^(Fresenius Medical Care), which is the focus of this study, belongs to this group of phosphate binders. This comparatively new preparation is an oral phosphate binder that contains two active compounds - calcium acetate (in reduced form), as well as magnesium carbonate (Composition: 435 mg calcium acetate (110 mg calcium) and 235 mg magnesium carbonate (60 mg magnesium)[12]) - which were shown to enable an effective, safe, and well-tolerated control of serum phos-phate levels in dialysis patients. The potential benefit of this therapy compared to calcium-based phosphate binders lies in the reduced daily intake of calcium combined with a higher phosphate-binding capacity: 1,000 mg OsvaRen^® ^has a theoretical phosphate-binding capacity of 58.4 mg phosphate, whereas 1,000 mg calcium acetate or calcium carbonate only bind 45 mg or 39 mg phosphate, respectively. The reduced daily intake of calcium reduces the risk of hypercalcaemia. However, a decisive advantage of a combination of calcium and magnesium should be the positive effect of magnesium on the heart and vessels. According to retrospective studies, magnesium potentially delays calcification of the vessels in dialysis patients, but this connection has not yet been evaluated prospectively. An investigation of the long-term effects of a calcium acetate/magnesium carbonate therapy has yet to be carried out. OsvaRen^® ^is currently licensed in 28 European countries for the therapy of hyperphosphataemia in conjunction with chronic renal failure in dialysis patients [4,12-14].

## Effectiveness and cost-effectiveness of OsvaRen^® ^vs. Renagel^® ^vs. calcium-containing phosphate binders

An ideal preparation - which is simultaneously effective, well-tolerated and, more-over, also cost-effective - does not currently exist: Aluminum-containing phosphate binders are very effective but, because of the danger of chronic intoxication, are no longer recommended for long-term treatment. Calcium acetate and calcium carbonate are most frequently used. They constitute an effective therapy for reducing the serum phosphate levels. Because of the additional daily supply of calcium and the increased risk of hypercalcaemia, they are discussed controversial. Sevelamer hydrochloride (Renagel^®^) and sevelamer carbonate (Renvela^®^) are the only metal(aluminium)-free phosphate binders. Due to the higher tablet burden, because of the frequent gastrointestinal complaints, and, in particular, as a result of higher costs, their use is also limited. Lanthanum carbonate (Fosrenol^®^) is likewise an expensive phosphate binder, as it is a very rare metal.

The effectiveness and tolerability of OsvaRen^® ^were recently demonstrated in a clinical study which was carried out in five European countries. Therefore, a comparison of currently existing information with respect to the effectiveness and cost-effectiveness for existing therapies for hyperphosphataemia and OsvaRen^® ^can be made. For this, published health economic evidence will be described and discussed against the background of the results of a new clinical study. Before an indirect comparison between calcium acetate/magnesium carbonate and sevelamer hydrochloride therapies is made (especially with respect to economic considerations) an overview of the comparative health economic studies which have been carried out up until now is given.

### The Huybrechts study for sevelamer

Two systematic reviews [6,15] are available for phosphate-binder therapy, both of which come to the conclusion that there is a lack of good studies of phosphate binders (especially long-term studies with a robust study design). In addition, the reviews come to the conclusion that sevelamer hydrochloride offers no benefit compared to its cost.

Hence, at this point, in particular, attention will be given to a study by Huybrechts et al [11] carried out in 2005. In the study, sevelamer was compared with calcium-containing preparations (calcium acetate and calcium carbonate) using a disease-simulation model. Over a time period of one year, the effects of cardiovascular conditions on life expectancy and the costs of medical care with sevelamer hydrochloride treatment were considered. The focus was to thereby evaluate non-inferiority of sevelamer hydrochloride therapy compared with calcium-containing preparations.

According to the results of this study, the calcification index fell within a year in the sevelamer group from 1,502 to 1,362 mg/dl, whereas, in contrast, the value in the calcium acetate group rose to 1,557 mg/dl. Accordingly, sevelamer hydrochloride treated patients (when compared to calcium treatment) had a reduced risk of calcification of 13% after one year. Also, the risk of severe cardiovascular events was about 12% lower compared to a calcium therapy. The study showed that, in a population of 100 patients, sevelamer prevented nine cardiovascular events, on average. This means that with eleven treated patients, one cardiovascular incident could be averted.

The most important component of overall costs of each therapy option was resource use due to inpatient treatment of cardiovascular events. In the sevelamer group, these represent about 74% and, in the calcium acetate group about 86% of overall costs. Huybrechts et al stated that the relatively higher costs of sevelamer therapy are almost offset by the prevention of nine cardiovascular events (which is associated with a saving of 200,000 USD in that study). This leads to cost-effectiveness ratio of less than 2,500 USD per (discounted) life year gained and about 4,500 USD per prevented cardiovascular event. The costs were estimated from a health insurance perspective on the basis of US specific resource consumption data. The methods are not described in further detail in the publication.

Although both investigated phosphate binders produced almost equivalent results with regard to the phosphate binding capabilities, Huybrechts et al concluded that the advantage of sevelamer potentially lies in its ability to slow down the process of cardiovascular calcification, from which the authors expect both positive clinical as well as economic effects.

In summary, the study therefore showed that sevelamer is not inferior compared to calcium therapy and, moreover, is effective in the prevention of cardiovascular events, the result of which is that overall costs could potentially be reduced. Finally, it should be mentioned that Huybrechts et al (2005) used a complex and modular model that follows individual patients via a discrete event simulation (DES) technique estimating both, one year as well as the long-term effects. The algorithms used were not completely disclosed in the publication.

## Studies of OsvaRen^®^

### OsvaRen^® ^vs. Renagel^® ^(CALMAG study)

The CALMAG (CALcium acetate-MAGnesium carbonate evaluation) study was a 24-week, randomized, controlled, multicenter parallel group study carried out in five European countries which compared the efficacy of the two phosphate binders: calcium acetate/magnesium carbonate (OsvaRen^®^) and sevelamer hydrochloride (Renagel^®^). The aim of the study was to determine whether OsvaRen^® ^therapy is equally effective in reducing the serum phosphate levels in haemodialysis patients as treatment with Renagel^® ^[12]. Of the 255 subjects who were included in the CALMAG study, 204 patients completed the trial. The remaining participants dropped out during the study for various reasons. The study population was divided into an OsvaRen^® ^group with 105 patients (drop outs n = 18) and a Renagel^® ^group with 99 patients (drop outs n = 34).

**Figure 1:**
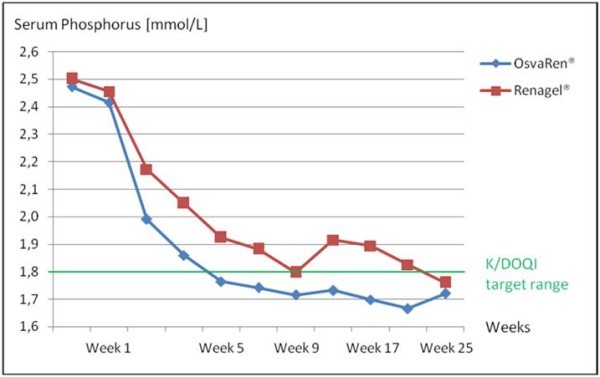
**Serum phosphate level **[9].

The results of the CALMAG study are presented in summary form in the next section. Figure 1 shows that a reduction in the serum phosphate levels was achieved with both preparations following a 24-week application. In the calcium acetate/magnesium carbonate group, the serum phosphate levels could be lowered to the clinically relevant K/DOQI target range already after four weeks of OsvaRen^® ^therapy whereas, in contrast, in the sevelamer hydrochloride group this value remained at a comparatively higher level beyond the total duration of the study and was first achieved in week 24. Furthermore, OsvaRen^® ^showed a significantly higher reduction in phosphate levels in the long-term and a better maintenance of serum phosphate levels in the K/DOQI target range was achieved.

Compared to the sevelamer hydrochloride group serum calcium levels in the calcium acetate/magnesium carbonate group were slightly increased over almost the whole time span, but the K/DOQI target value was at no time exceeded. In addition, with regard to the ionised serum calcium, there was no significant difference between the calcium acetate/magnesium carbonate and the sevelamer hydrochloride group. Hence, with regard to the control of the serum calcium levels and the risk of hypercal-caemia, no clinically relevant difference between the calcium acetate/magnesium carbonate group and the sevelamer hydrochloride group was reported. Following these outcomes, it can be concluded that treatment with calcium acetate/magnesium carbonate does not lead to an increased risk of hypercalcaemia compared to sevelamer hydrochloride.

Serum magnesium levels in the calcium acetate/magnesium carbonate group were as expected slightly increased. Symptoms associated with raised magnesium levels, however, did not appear during the study. In adults, the normal level for serum magnesium ranges between 0.7 and 1.1 mmol/l such that, especially in the case of haemodialyis patients, there is frequently a slight increase of these values. In recent years, a number of studies have shown that higher and increased serum magnesium levels in dialysis patients are associated with positive cardiovascular characteristics, such as, an anti-calcification effect as well as improved survival [16-22]. According to the label information, OsvaRen^® ^is only contraindicated in patients with a serum magnesium level of more than 2 mmol/l.

Therefore, the CALMAG study came to the conclusion that, with regard to the primary endpoint - i.e. the reduction in the phosphate levels; - OsvaRen^® ^is at least as effective as Rena-gel^®^. Moreover, OsvaRen^® ^was shown to be superior in achieving and maintainting levels of serum phosphate within the K/DOQI target range and proved to be comparable in maintaining serum calcium levels within the K/DOQI target range. Finally, with OsvaRen^®^, gastrointestinal complaints were reduced by about 50% compared with Renagel^® ^[12].

### OsvaRen^® ^vs. calcium-containing phosphate binders - the Deuber study

Because of the addition of magnesium, which also acts as a phosphate binder, OsvaRen^® ^also shows an improved phosphate-binding capacity compared to pure calcium-containing phosphate binders in combination with a reduced daily calcium intake. Compared to a calcium acetate tablet of 660 mg (corresponding to 167 mg elemental calcium), OsvaRen^® ^as a combination preparation contains 35% less elemental calcium. However, as a consequence of its magnesium component, OsvaRen^® ^has a 30% higher phosphate-binding capacity per film tablets compared to pure calcium acetate (39.1 mg vs. 29.7 mg). Compared to a calcium carbonate tablet of 500 mg (corresponding to 200 mg elemental calcium), calcium acetate/magnesium carbonate (following conversion to a comparable dose of 670 mg) contains 59% less elemental calcium. At the same time the combination formulation has, because of the magnesium component, a 50% higher phosphate-binding capacity per film tablet than pure calcium carbonate (39.1 mg vs. 19.5 mg) [14].

The advantage of a calcium acetate/magnesium carbonate therapy compared to calcium-containing phosphate binders was established already in 2004 in a 36-month randomized, controlled clinical study [23] in which 50 haemodialysis patients were subjected to either a calcium carbonate monotherapy or a combination preparation consisting of calcium acetate/magnesium carbonate. The focus of that study was the change in the serum calcium, serum phosphate, and serum magnesium levels over the course of time. The data obtained from the study demonstrated that the combination preparation produced a significant and sustained reduction in both the phosphate level in the blood, and the serum calcium concentration. In contrast, there was an increase in the serum magnesium level which, however, did not exceed the normal value for adults and therefore was not critical.

Deuber was therefore able to demonstrate that the combination of calcium acetate and magnesium carbonate is superior in reducing the serum phosphate levels compared to calcium monotherapy, particularly since OsvaRen^® ^can be given in higher doses without causing any relevant increase in serum calcium [23]. Due to the better long-term control of the serum calcium level, OsvaRen^® ^contributes to a reduction in the frequency of hypercalcaemic episodes and thus also the associated subsequent costs [24].

## Conclusions

OsvaRen^® ^offers an innovative preparation for effective long-term treatment of a raised serum phosphate level in patients with chronic kidney failure. From the clinical perspective, OsvaRen^® ^is at least as effective in terms of phosphate binding as sevelamer hydrochloride (Renagel^®^) and is superior compared to calcium-containing preparations. The analyses of the CALMAG and Deuber studies confirm the superiority of the combination preparation. OsvaRen^® ^therefore should be preferred to a calcium carbonate treatment, as it is more effective in achieving the K/DOQI target values for serum phosphate and especially serum calcium, and thereby reducing the risk of hypercalcae-mia.

Although OsvaRen^® ^and Renagel^® ^achieved similar results in reaching the primary endpoint - i.e. the reduction of serum phosphate levels - there are some arguments that OsvaRen^® ^might offer a valid therapeutic alternative versus Renagel^®^: The evidence has shown that when OsvaRen^® ^is compared with Renagel^® ^the target range for serum phosphate defined in the K/DOQI guidelines can more rapidly be achieved. In addition, it is equally good at achieving the levels for serum calcium and therefore, in summary, to act preventatively with regard to the excess morbidity and mortality of haemodialysis patients.

In addition to the positive effect of the magnesium component, the high phosphate binding capacity and the ability to slow the possible mechanism of the vessel calcification process OsvaRen^® ^therapy has a decisive influence on the overall costs associated with these therapeutic options. Because of the price difference between OsvaRen^® ^and Renagel^® ^(OsvaRen^® ^has about 85% lower acquisition costs), the conclusions from the Huybrecht study regarding the economic advantages of Renagel^® ^treatment from the perspective of the health insurance have to be reconsidered with the data for OsvaRen^®^, which was not included in that analysis.

**Figure 2:**
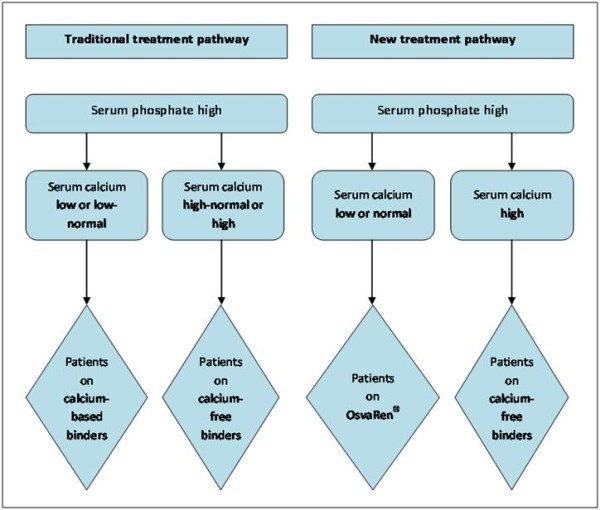
**Possible optimal treatment pathway**.

Figure 2 shows in schematic form how OsvaRen^® ^might be optimally included in phosphate binding therapy. Because of its clinical properties offering an at least non-inferior clinical profile combined with an economic superiority, OsvaRen^® ^can be recommended as a cost-effective, first-line treatment both for patients who receive a phosphate binder for the first time, as well as also for all patients who, up until now, have received a pure calcium-based phosphate binder or calcium-free phosphate binders.

Within the German health care setting, the benefit to a patient is considered to be an improvement in the state of health, a reduction in the duration of the illness, an increase in life time, a reduction in side-effects, as well as an improvement in the quality of life. From the perspective of the Institute for Quality and Efficiency in Health Care (IQWiG), this corresponds to a (positive) impact on the primary outcome pa-rameters (combined with a safe risk-benefit profile):

• mortality and

• morbidity and

• health-related quality of life.

OsvaRen^®^, in this respect, is able to fulfil the target parameters of the IQWiG, because of the reduction in adverse drug reactions (reduced risk of hypercalcaemia) as well as an improvement in the health-related quality of life. Because of the efficacy and tolerability profile of OsvaRen^®^, the preparation is a medically suitable and, at the same time, a cost-effective solution for the therapy of hyperphosphataemia. Therefore, it can be recommended to use OsvaRen^® ^as a first-line treatment option in the treatment of hyperphosphataemia.

## Abbreviations

IQWiG: Institute for Quality and Efficiency in Health Care.

## Competing interests

This work was supported by Fresenius Medical Care Deutschland GmbH, Bad Homburg, Germany. Apart from the funding, the authors declare to have no further conflict of interest.

## Authors' contributions

TP: Literature search and writing of the manuscript. AP, TM: Critical review of the manuscript. All authors read and approved the final draft
